# Growing oocyte-specific transcription-dependent de novo DNA methylation at the imprinted *Zrsr1*-DMR

**DOI:** 10.1186/s13072-018-0200-6

**Published:** 2018-06-06

**Authors:** Keiichiro Joh, Fumikazu Matsuhisa, Shuji Kitajima, Kenichi Nishioka, Ken Higashimoto, Hitomi Yatsuki, Tomohiro Kono, Haruhiko Koseki, Hidenobu Soejima

**Affiliations:** 1 Division of Molecular Genetics and Epigenetics, Department of Biomolecular Sciences, Faculty of Medicine, Saga University, Saga, 849-8501 Japan; 2 Division of Biological Resources and Development, Analytical Research Center for Experimental Sciences, Saga University, Saga, 849-8501 Japan; 3 Laboratory of Animal Developmental Biology, Department of Bioscience, Faculty of Applied Biosciences, Tokyo University of Agriculture, Tokyo, 156-8502 Japan; 4 Laboratory for Developmental Genetics, RIKEN Center for Integrative Medical Science, Yokohama, Kanagawa 230-0045 Japan

**Keywords:** DMR, DNA methylation, Genomic imprinting

## Abstract

**Background:**

*Zrsr1* is a paternally expressed imprinted gene located in the first intron of *Commd1*, and the *Zrsr1* promoter resides in a differentially methylated region (DMR) that is maternally methylated in the oocyte. However, a mechanism for the establishment of the methylation has remained obscure. *Commd1* is transcribed in the opposite direction to *Zrsr1* with predominant maternal expression, especially in the adult brain.

**Results:**

We found *Commed1* transcribed through the DMR in the growing oocyte. *Zrsr1*-DMR methylation was abolished by the prevention of *Commd1* transcription. Furthermore, methylation did not occur at the artificially unmethylated maternal *Zrsr1*-DMR during embryonic development when transcription through the DMR was restored in the zygote. Loss of methylation at the maternal *Zrsr1*-DMR resulted in biallelic *Zrsr1* expression and reduced the extent of the predominant maternal expression of *Commd1.*

**Conclusions:**

These results indicate that the establishment of methylation at *Zrsr1*-DMR occurs in a transcription-dependent and oocyte-specific manner and caused *Zrsr1* imprinting by repressing maternal expression. The predominant maternal expression of *Commd1* is likely caused by transcriptional interference by paternal *Zrsr1* expression.

**Electronic supplementary material:**

The online version of this article (10.1186/s13072-018-0200-6) contains supplementary material, which is available to authorized users.

## Background

Genomic imprinting is an epigenetic phenomenon of parent-of-origin-dependent expression that is observed in a subset of mammalian genes. Imprinted genes are expressed exclusively or predominantly from one of the two parental alleles and are frequently located in clusters known as imprinted domains. The expression of genes in an imprinted domain is regulated by a discrete element called an imprinting center (IC) or an imprinting control region (ICR). Imprinted genes or imprinted domains are associated with differentially methylated regions (DMRs) that exhibit parent-of-origin-specific DNA methylation. Two classes of DMRs have been identified as follows: germline DMRs (gDMRs), or primary DMRs; and somatic DMRs (sDMRs), or secondary DMRs. gDMR methylation is established during gametogenesis, and sDMRs acquire methylation after fertilization under the direction of gDMRs. The ICs of the imprinted genes are located in their corresponding gDMRs. More than 20 gDMRs have been identified in mice, of which only three are paternally methylated [1–4]. Recent studies have identified an additional 11 new putative maternally methylated gDMRs [5].

DNA methylation at gDMRs is the primary determinant of the allelic expression of imprinted genes, and the mechanisms of methylation establishment have been extensively investigated. The specific recruitment of de novo methylation machineries to gDMR methylation sites via the recognition of sequence elements and/or chromatin structures has been considered as a potential mechanism of germline-specific gDMR methylation establishment [1, 6]. However, efforts to identify sequence motifs for gDMR methylation have not been successful. Several trans-acting factors for maternally methylated gDMRs have been found to be essential for the establishment of germline methylation in mice. For example, Dnmt3a has been identified as the enzyme responsible for de novo methylation of many maternal gDMRs [7, 8]. Dnmt3l, a DNA methyltransferase (DNMT)-like protein without enzymatic activity, is the likely co-factor of DNMTs [9]. Ablation of Kdm1b, a histone demethylase of H3K4 di-and trimethylation, in oocytes resulted in the failure of methylation establishment at some maternal gDMRs [10]. In addition, the deletion of *Hira*, which encodes a histone H3.3 chaperon (Hira), led to global hypomethylation in oocytes [11].

Kelsey et al. proposed a model for the establishment of methylation at the maternal gDMRs in the oocyte [12, 13], which suggests that maternal methylation of gDMRs is regulated by the same mechanisms of general gene-body methylation reported for active genes [14, 15]. This was based on the findings that most maternal gDMRs are located in actively transcribed regions [16], that transcription is a prerequisite for the establishment of methylation at four maternal gDMRs [13, 16–18], and regarding the characteristics of the methylome and transcriptome in growing oocytes [13, 19]. Analyses of the methylome revealed that most methylation in the oocyte genome occurs within actively transcribed regions and that maternal gDMRs are not specifically targeted for methylation, but are instead methylated along with other parts of the transcribed regions where the gDMRs reside. Unlike the rest of the transcribed regions, gDMRs are likely protected against global demethylation during early embryonic development; only the gDMRs escape global demethylation and remain methylated throughout development.

Methylation failed to be established at the gDMRs in the *Gnas* locus and the KvDMR in the *Kcnq1* locus when a poly(A) signal truncation cassette was inserted into these loci to prevent transcription from elongation through the gDMRs [16, 18]. Failure of methylation was also reported at PWS-IC and the *Zac1*-DMR when the promoter regions from which transcription originated and then proceeded through the maternal gDMRs were deleted [13, 17]. Furthermore, most of these maternal gDMRs were located within transcribed regions in the growing oocyte [16]. On the other hand, the establishment of sDMR- and lineage-specific methylation during the post-implantation stage clearly indicates the de novo methylation potency of early embryonic cells, including naïve/primed pluripotent stem cells [20]. However, it is unknown whether de novo methylation occurs at gDMRs during the post-implantation stage after failure to establish gDMR methylation in the oocyte.

*Zrsr1* (*U2af1*-*rs1*) and *Commd1* (*Murr1*) are imprinted protein coding genes located in mouse proximal chromosome 11. *Zrsr1* is expressed ubiquitously in all adult tissues examined and is expressed exclusively from the paternal allele. *Zrsr1* resides in the first intron of the *Commd1* gene and is transcribed in the opposite direction to the host gene. The *Zrsr1* promoter is located in a maternally methylated gDMR, i.e., the *Zrsr1*-DMR [21]. It is likely that maternal methylation at the *Zrsr1*-DMR causes imprinted expression by repressing maternal expression of the gene. *Commd1* is likewise expressed ubiquitously in adult mice, but is expressed from both parental alleles. However, *Commd1* expression from the maternal allele is stronger than expression from the paternal allele (i.e., predominant maternal expression), as exemplified in the adult brain. Although *Zrsr1*-DMR resides in the transcribed region of *Commd1*, the link between transcription and the establishment of methylation at this DMR has not been clarified.

In this study, we found that methylation at *Zrsr1*-DMR failed to be established when transcription through the DMR was abolished by the insertion of a poly(A) signal cassette into the site between the *Commd1* promoter and the *Zrsr1* gene. Furthermore, upon deletion of the cassette in the zygote, *Zrsr1*-DMR transcription resumed, but methylation at the DMR during early development was not restored. These results indicate that transcription-dependent methylation at the DMR occurs specifically in the growing oocyte, but not during early development. We also found that interference with transcription likely caused predominant maternal expression of *Commd1* in the adult brain.

## Results

### Truncation of *Commd1* transcription results in methylation failure at *Zrsr1*-DMR in the growing oocyte

**Fig. 1:**
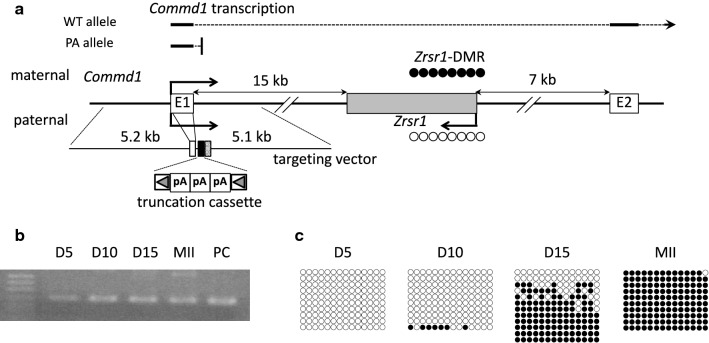
Structure of the *Zrsr1*/*Commd1* locus and analysis of *Commd1* expression and *Zrsr1*-DMR methylation in the oocyte. **a** *Zrsr1*, an approximately 2.8-kb intronless gene, and the first two exons of *Commd1* are represented by gray and white boxes, respectively. Distances from the *Zrsr1* gene to *Commd1* exon 1 and exon 2 are indicated above the gene with double-headed arrows. The schematic is not drawn to scale. Arrows above (maternal allele) and below (paternal allele) exon 1 and the *Zrsr1* gene represent the direction of transcription and the allelic expression status of the genes. The open and closed circles at the *Zrsr1* promoter indicate unmethylation and methylation, respectively. A schematic of the targeting vector is shown under the gene. The closed and hatched boxes represent the truncation cassette and the neo-selection marker gene, respectively. These elements are flanked by the 5.2 kb left arm containing exon 1 and the 5.1 kb right arm, which contains part of intron 1. The truncation cassette is flanked by loxP sites, represented by gray arrowheads enclosed in open rectangles. Expected transcription patterns of the WT and PA alleles are shown above the gene schematic with thick lines and dotted lines corresponding to exons and introns, respectively. **b** RT-PCR analysis of *Commd1* expression in growing oocytes prepared from B6 female neonates at Day 5 (D5), Day 10 (D10), and Day 15 (D15) postpartum, and fully grown MII oocytes (MII) from B6 adult females. PC: positive control for RT-PCR using adult brain cDNA. MW: molecular weight marker. **c** Analysis of methylation at *Zrsr1*-DMR in growing and fully grown oocytes used in **b**. The 223-bp region in the DMR containing 14 CpGs was analyzed via bisulfite sequencing. Each row represents a dataset from one clone, and each circle represents one CpG site. Closed and open circles depict methylated and unmethylated CpGs, respectively

Methylation of maternal gDMRs of imprinted genes is established asynchronously during postnatal oocyte growth, typically between 5 and 25 days postpartum (dpp) [22]. To test whether de novo methylation at *Zrsr1*-DMR is dependent on transcription resulting from *Commd1* expression in the oocyte, we analyzed *Commd1* expression and the methylation status of *Zrsr1*-DMR in growing oocytes from this period and in ovulated MII oocytes. *Commd1* was expressed in all periods of oocyte maturation analyzed (Fig. 1b). De novo methylation at *Zrsr1*-DMR started after 10 dpp and was completed between 15 dpp and maturation (Fig. 1c). Thus, transcription through the *Zrsr1*-DMR proceeded before and during the establishment of methylation. *Zrsr1* expression was not detected by RT-PCR during oocyte maturation (Additional file 1: Figure S1).

To determine whether de novo methylation at *Zrsr1*-DMR was dependent on *Commd1* elongation through this DNA element, we inserted a truncation cassette containing three tandem copies of SV40 poly(A) signal into intron 1 of *Commd1* to generate the transcription-truncation allele *Commd1*^*PA*^ (Fig. 1a) and obtained *Commd1*^+*/PA*^-heterozygous mice in a C57BL/6 J background. No *Commd1*^*PA/PA*^ mice were born from the intercrossing of heterozygous parents. *Commd1*^−*/*−^ mice have been shown to be embryonically lethal at E9.5 to E10.5 [23]; the absence of homozygous pups for the truncation allele was thus likely attributable to embryonic lethality, which strongly suggests that a truncation occurred as expected and rendered the *Commd1*^*PA*^ allele functionally null.

**Fig. 2:**
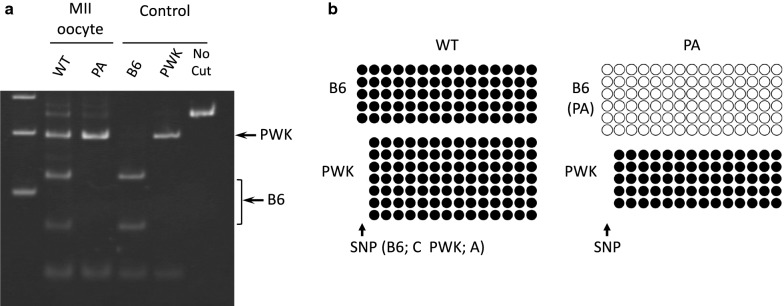
Analysis of allelic *Commd1* expression and *Zrsr1*-DMR methylation in PA and WT MII oocytes. **a** MII oocytes were prepared from *Commd1*^*PA(B6)/*+*(PWK)*^ females (PA) and *Commd1*^+*(B6)/*+*(PWK)*^ females (WT). RT-PCR was performed as in Fig. 1b. Amplified cDNA (250 bp) was digested with NlaIII (CATG). There are two NlaIII restriction sites in the B6 amplicon, but one of these is absent in the PWK amplicon because of a single nucleotide polymorphism (SNP) between the two strains. Adult brain RNA from each of the strains was used as the control. MW: molecular weight marker. **b** A 278-bp region in *Zrsr1*-DMR containing 15 CpG sites (B6) or 14 CpG sites (PWK) was analyzed via bisulfite sequencing. The alleles were discriminated via an SNP (C in B6 and A in PWK) located in the leftmost CpG site in the B6 sequence. Closed and open circles depict methylated and unmethylated CpGs, respectively

To assess the truncation of the *Commd1*^*PA*^ allele and *Zrsr1*-DMR methylation in the MII oocyte, MII oocytes were obtained from adult F1 females generated from the cross between *Commd1*^+*/PA*^ B6 females and WT PWK males. *Commd1*^*PA(B6)/*+*(PWK)*^ mice, termed PA F1 mice, and *Commd1*^+*(B)/*+*(PWK)*^ mice, termed WT F1 mice, were obtained from F1 littermates. The allelic expression of *Commd1* was analyzed by RFLP analysis of RT-PCR products with the primers Comm-F1 and Comm-R1, located at exon 1 and exon 2, respectively. Expression of the *Commd1*^*PA*^ allele was not detected in MII oocytes prepared from the PA F1 females, although expression of the PWK allele was detected. In contrast, both alleles were expressed in oocytes from WT F1 female littermates (Fig. 2a). The *Zrsr1*-DMR was completely unmethylated on the truncated allele (B6) in the MII oocytes from the PA F1 females, in contrast to the WT PWK allele, which was completely methylated. As expected, in oocytes from the WT F1 females, *Zrsr1*-DMR was completely methylated on both the B6 and PWK alleles (Fig. 2b). These results indicate that transcription termination occurred in intron 1, likely at the truncation cassette, and resulted in the loss of transcription through the DMR, which led to methylation failure at the DMR during oogenesis.

### Maternal methylation at *Zrsr1*-DMR causes *Zrsr1* imprinting

**Fig. 3:**
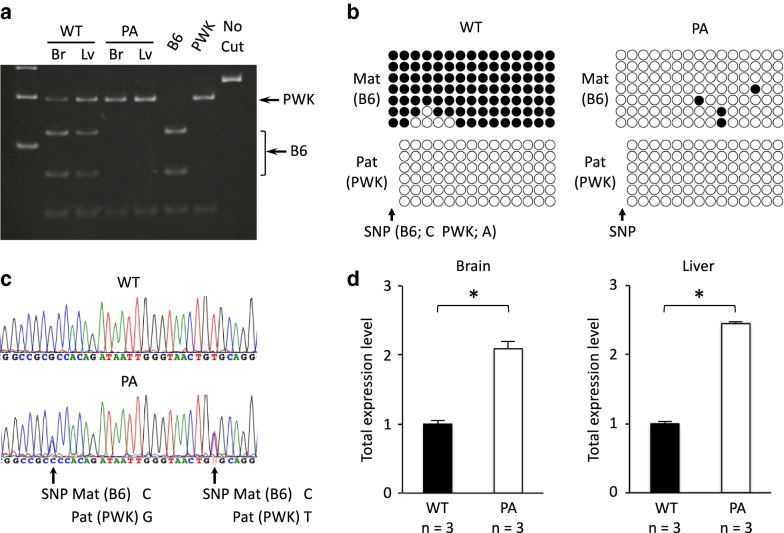
Analysis of *Commd1* and *Zrsr1* expression and *Zrsr1*-DMR methylation in adult mice. **a** Allelic expression of *Commd1* was analyzed as in Fig. 2a in the brain (Br) and liver (Lv) of *Commd1*^*PA(B6)/*+*(PWK)*^ (PA) and *Commd1*^+*(B6)/*+*(PWK)*^ (WT) adult F1 mice. MW: molecular weight marker. B6 and PWK are the maternal and paternal alleles in the F1 mice, respectively. **b** Methylation at *Zrsr1*-DMR was analyzed as in Fig. 2b in the brain of adult F1 mice. Shown is the SNP used to discriminate the parental alleles. **c** Allelic expression of *Zrsr1* was analyzed in the F1 adult brain by direct RT-PCR sequencing of the cDNA, which contains two SNP sites between B6 and PWK alleles. **d** Total *Zrsr1* expression was analyzed in triplicate (shown with n) in the adult F1 brain and liver via TaqMan RT-PCR. Total expression is presented relative to the WT expression level in each tissue. The differences in levels of expression between WT and ΔPA F1 mice were statistically significant in the brain and liver (two-sided Student’s *t* test, *P < 0.01)

To investigate the causative link between the maternal methylation at *Zrsr1*-DMR and the imprinted expression of *Zrsr1*, we analyzed *Zrsr1*-DMR methylation and *Zrsr1* allelic expression in the adult F1 mice described above. The truncation of *Commd1* transcription was also observed in the brain and liver of the PA F1 mice (Fig. 3a). The *Zrsr1*-DMR on the maternal *Commd1*^*PA(B6)*^ allele of the PA F1 mice was completely unmethylated, whereas normal maternal methylation was observed in the WT F1 mice (Fig. 3b). These results indicate that the truncation cassette was also functional in the adult mice and that the unmethylated status of maternal *Zrsr1*-DMR persisted throughout the embryo stage and postnatal growth to adulthood. *Zrsr1* was biallelically expressed in the PA F1 mice, while in WT F1 mice the gene exhibited paternal-specific expression in the WT F1 mice (Fig. 3c). Quantitative analysis of total *Zrsr1* mRNA levels indicated that the PA F1 mice expressed twice as much *Zrsr1* as the WT F1 mice (Fig. 3d), which suggests that the maternal allele in the PA F1 mice was completely de-repressed and was expressed at the same level as the paternal allele. Thus, it is evident that the *Zrsr1* gene is imprinted by suppression of maternal allele expression and that maternal methylation at the DMR is the primary imprint mark.

### Zygotic deletion of the truncation cassette does not result in the acquisition of methylation at *Zrsr1*-DMR during embryonic development and postnatal growth

**Fig. 4:**
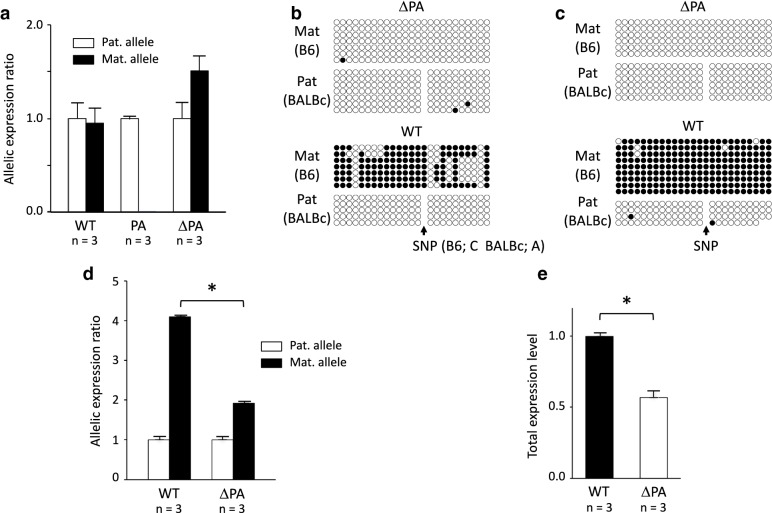
Quantitative analysis of *Commd1* expression and *Zrsr1*-DMR methylation in ΔPA blastocysts and the ΔPA adult brain. **a** Allelic expression of *Commd1* was analyzed in *Commd1*^+*(B6)/*+*(BALB)*^ (WT), *Commd1*^*PA(B6)/*+*(BALB)*^ (PA), and *Commd1*^*ΔPA(B6)/*+*(BALB)*^ (ΔPA) blastocysts in triplicate (shown with n) by pyrosequencing. Alleles were discriminated between using an SNP between B6 (Mat) and BALB/c (Pat) located in exon 2 (rs26846230; C in B6, T in BALB/c). The allelic expression ratios are presented relative to paternal expression in each blastocyst sample. **b** Methylation at *Zrsr1*-DMR was analyzed in the blastocysts used in **a**. The 343-bp region in the DMR containing 25 CpG sites (B6; maternal allele) or 24 CpG sites (BALB/c; paternal allele) was analyzed via bisulfite sequencing. The alleles were discriminated between using an SNP (rs26846192; C in B6, A in BALB/c) located in a CpG site in the B6 sequence. Closed and open circles depict methylated and unmethylated CpGs, respectively. **c** Methylation at *Zrsr1*-DMR was analyzed in the brains of ΔPA and WT adult F1 mice as described in **b**. **d** Allelic expression of *Commd1* in the adult F1 brain was quantitatively analyzed in triplicate (shown with n) by pyrosequencing and is presented as in **a**. **e** Total *Commd1* expression in an adult F1 brain of each genotype was analyzed in triplicate (shown with n) via TaqMan RT-PCR. Total expression is presented relative to the WT expression level. Asterisks (*) indicate statistical significance (*P* < 0.01) according to the two-sided Student’s *t* test

We showed that *Zrsr1*-DMR acquires methylation in a transcription-dependent manner during oogenesis. However, *Commd1* is expressed from both alleles and paternal *Zrsr1*-DMR is unmethylated in various tissues of adult mice [21, 24]. This fact indicates that transcription-dependent methylation does not occur at paternal *Zrsr1*-DMR in differentiated somatic cells. However, it was not known that *Commd1* is expressed in early embryo, in which genome-wide de novo methylation is about to occur [19, 25, 26]. *Commd1* was expressed from both alleles in the WT blastocysts described below (WT in Fig. 4a, b), which indicated that the paternal *Zrsr1*-DMR was also unmethylated, even during transcription in pluripotent cells. To confirm this in the maternal allele, the floxed truncation cassette (refer to Fig. 1a) was deleted from the maternal *Commd1*^*PA*^ allele via zygotic expression of Cre recombinase from CAG-Cre transgene. Then, methylation at the maternal *Zrsr1*-DMR, which was inherited in an unmethylated state, was analyzed. The resulting deleted allele was denoted *Commd1*^*ΔPA*^.

The blastocysts were prepared via in vitro fertilization (IVF) with MII oocytes from the *Commd1*^+*/PA*^ B6 females and sperm from CAG-Cre transgenic males in a BALB/c background. Deletion of the truncation cassette occurred in all blastocysts that inherited the *Commd1*^*PA*^ allele and the CAG-Cre transgene(s) (Additional file 2: Figure S2). Judging from the high incidence of deletion at the blastocyst stage, it is plausible that the deletion actually occurred much earlier. To determine whether deletion of the truncation cassette would restore expression of the maternal *Commd1* allele, we analyzed the allelic expression of *Commd1* in *Commd1*^+*(B6)/*+*(BALB)*^ blastocysts (WT), *Commd1*^*PA(B6)/*+*(BALB)*^ blastocysts (PA), and *Commd1*^*ΔPA(B6)/*+*(BALB)*^ blastocysts (ΔPA). Allelic expression was analyzed quantitatively by pyrosequencing of RT-PCR products with the primers CommExpre-F1-bio and CommExprePyro-R (Table S1), which are located in exon 2. In the WT blastocysts, *Commd1* was equally expressed from both parental alleles, but only the paternal allele was expressed in the PA blastocysts. This indicates that the truncation cassette was also functional in the blastocysts, and suggests that *Commd1* was expressed from both alleles during the developmental period in which global de novo DNA methylation occurs. In ΔPA blastocysts, in which the truncation cassette was deleted, the maternal ΔPA allele was expressed at levels comparable to the paternal allele (Fig. 4a). Furthermore, the unmethylated status of maternal *Zrsr1*-DMR was maintained, despite transcription of the DMR (Fig. 4b). To determine whether this status was maintained while under transcription from the early embryonic stage to adulthood, we analyzed the brain of ΔPA adult F1 mice from the cross between *Commd1*^+*/PA*^ B6 females and CAG-Cre transgenic BALB/c males. The maternal *Zrsr1*-DMR in the *Commd1*^*ΔPA*^ allele was unmethylated, even though the maternal allele was expressed (Fig. 4c, d). Thus, we concluded that maternal *Zrsr1*-DMR was not methylated after fertilization, irrespective of DMR transcription.

### Predominant maternal expression of *Commd1* is caused by the imprinted expression of *Zrsr1*

**Fig. 5:**
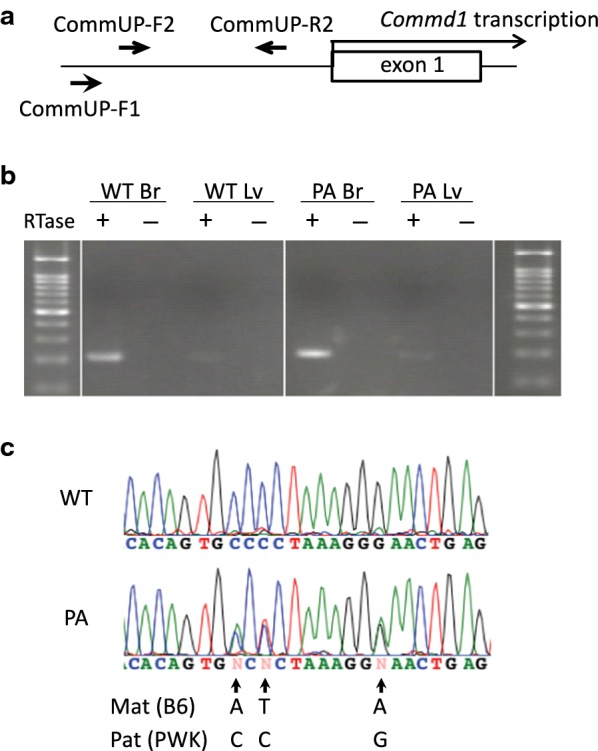
The antisense transcript in the promoter region of *Commd1* in adult tissues. **a** Strand-specific RT-PCR was performed to detect an antisense transcript to *Commd1* transcription in the promoter region. The arrows represent the primers, CommUP-F1, for cDNA synthesis, and CommUP-F2 and CommUP-R2, for subsequent RT-PCR. The amplicon is 206 bp in size. CommUP-R2 is located approximately 100-bp upstream from the putative transcription start site of *Commd1*. **b** Brain (Br) and liver (Lv) RNA were analyzed. Tissues were prepared from adult F1 mice generated from crossing PA-B6 females with WT PWK males. cDNA synthesis was performed with (RTase +) or without (RTase −) reverse transcriptase. MW: molecular weight marker. **c** Allelic expression of the antisense transcripts was analyzed via direct sequencing of the amplified brain cDNA from the WT and PA mice shown in **b**. Three SNPs used to discriminate the parental alleles are shown under the electropherograms

We reported predominant maternal expression of *Commd1*, especially in the brains of adult mice [24] (WT in Fig. 4d, Additional file 3: Figure S3). Based on the two observations [24] discussed below, we hypothesized that the expression of *Zrsr1* decreased *Commd1* expression from the paternal allele by interfering with the transcription elongation of *Commd1* within the gene body and with transcription initiation in the *Commd1* promoter. First, in the adult liver, *Zrsr1* is expressed at extremely low levels (less than 10%) relative to *Commd1*, which exhibits less pronounced predominant maternal expression (Additional file 3: Figure S3). However, in the adult brain, *Zrsr1* is expressed to the same extent as *Commd1*, which exhibits remarkable predominant maternal expression. Second, the antisense transcript of *Commd1* was observed in the promoter region of *Commd1* only in the paternal allele and was observed at higher levels in the brain than in the liver (also refer to [27] and Fig. 5b). The observed antisense *Commd1* transcript is presumably generated by read-through transcription of *Zrsr1*, which may interfere with the initiation of *Commd1* transcription.

According to this hypothesis, we expected that predominant maternal expression would not occur in the ΔPA mice, which express *Zrsr1* equally from both parental alleles. Indeed, quantitative analysis by pyrosequencing demonstrated that the ratio of maternal to paternal expression was significantly decreased in these mice relative to WT mice (Fig. 4d). The total *Commd1* expression levels were then quantitatively analyzed in both lines using the TaqMan assay (Fig. 4e). Expression of *Commd1* in the ΔPA mice was 57% of that in the WT mice, which is consistent with the notion of decreased maternal expression in the ΔPA mice. Moreover, the relative quantities of the overall expression were consistent with the quantitative allelic expression analysis shown in Fig. 4d, in which the total expression levels were 5 (maternal = 4, paternal = 1) and 3 (maternal = 2, paternal = 1) in the WT mice and the ΔPA mice, respectively, given no change in the level of expression from the paternal allele. Collectively, these results indicate that expression of *Zrsr1* reduces the expression of *Commd1* from the paternal allele and results in the predominant maternal expression of *Commd1*.

In the *Commd1* promoter region, we analyzed antisense transcription of *Commd1* in F1 mice generated from the cross between *Commd1*^+*/PA*^ B6 females and WT PWK males. Antisense transcripts were expressed at higher levels in the brain than in the liver of the WT mice, as reported previously [24], and the same transcript was observed in the PA mice (Fig. 5b). In addition, the antisense transcript was expressed only from the paternal allele in the WT mice, which is congruent with previous studies [24]. However, it was expressed from both alleles in the PA mice (Fig. 5c), which was consistent with the pattern of allelic expression of *Zrsr1* in these mice. This consistency of allelic expression between the opposite transcripts and *Zrsr1* strongly supports the idea that to some degree the transcription of *Zrsr1* extends to the *Commd1* promoter region beyond the poly(A) signal of the *Zrsr1* gene. Such antisense transcription might interfere with the initiation of *Commd1* transcription and thereby contribute to its predominant maternal expression, especially in the brain.

## Discussion

The results of this study indicate that transcription through the *Zrsr1*-DMR is essential for the establishment of methylation at the DMR in the growing oocyte, as reported for four other imprinted loci [13, 16–18]. Our results also indicate that transcription-dependent methylation in the *Zrsr1*-DMR does not occur after fertilization, which suggests that the mechanism of de novo methylation of the *Zrsr1*-DMR is oocyte-specific. We also found that paternally imprinted expression of *Zrsr1* resulted in predominant maternal expression of *Commd1*, which is likely caused by transcriptional interference between *Zrsr1* and *Commd1* on the paternal allele.

The mouse embryo undergoes genome-wide de novo DNA methylation during the post-implantation period, at a level comparable to the growing oocyte [19, 25, 26]. In fact, the early embryo expresses factors essential for de novo DNA methylation, such as Dnmt3a, Dnmt3b, and Dnmt3l [28–30]. In this respect, the de novo DNA methylation activity in the pluripotent cells of the early embryo is comparable to that in the growing oocyte. However, our results indicated that methylation at *Zrsr1*-DMR does not occur on either parental allele in the early embryo, despite *Commd1* expression from both alleles (Fig. 4a–c). This suggests some differences in the mechanisms of de novo methylation between the early embryo and the growing oocyte, at least in *Zrsr1*-DMR. Two possible differences are considered: first, there may be factor(s) inhibiting de novo methylation at *Zrsr1*-DMR in the early embryo, e.g., histone modification(s) at the DMR, or a trans-acting factor(s) expressed in pluripotent cells; second, the early embryo may lack factor(s) required for de novo methylation at the DMR, which could include epigenetic modification(s) at the DMR that are erased after fertilization, or trans-acting oocyte-specific factor(s). In contrast to the methylation at *Zrsr1*-DMR, transcription-dependent methylation occurs during embryonic development at sDMRs located upstream of *Zdbf2*, which is an imprinted gene on the paternal allele, during the period around implantation [31]. This suggests variation in DNA methylation mechanisms among DMRs in the imprinted loci, and it will be interesting to examine whether transcription-dependent methylation is also oocyte-specific at the four previously reported loci.

Several mechanisms are known or proposed for imprinted gene silencing, such as by allele-specific inactivation of an enhancer–insulator residing in a gDMR and by recruitment of repressive chromatin modifiers through a long non-coding RNA with imprinted expression [2, 4]. Methylation of a gene promoter usually represses expression of the respective gene [32, 33]. Thus, the paternal allele-specific expression of *Zrsr1* seems to be caused by maternal methylation at *Zrsr1*-DMR. This was confirmed by analyses of the expression and methylation of *Zrsr1* in the PA mice, in which the absence of methylation at the maternal *Zrsr1*-DMR resulted in equal levels of gene expression from both alleles (Fig. 3c, d). A decrease in maternal expression of *Commd1* also occurred concomitantly with the activation of maternal *Zrsr1*, which strongly suggests that the predominant maternal expression of *Commd1* results from *Zrsr1*-mediated reduction in the expression of *Commd1* from the paternal allele. Studies on genetic organization in genomes have identified overlapping and antisense-oriented genes, and the mutual *cis*-acting effect on their expression, which is termed transcriptional interference [34]. The active transcription of *Zrsr1* within *Commd1* may cause collisions between the opposing *Zrsr1* and *Commd1* elongation complexes, which would result in the reduction in their expression. We observed an antisense-oriented transcript in the *Commd1* promoter region, which was hypothesized to be an overshoot of *Zrsr1* transcription (Fig. 5) and suggests that *Zrsr1* transcription potentially interferes with the initiation of *Commd1* transcription. However, complete biallelic *Zrsr1* expression did not give rise to equal levels of *Commd1* expression from both parental alleles (Fig. 4d). Therefore, unidentified differences between the parental *Commd1* alleles may exist, despite the fact that thus far we have found no differences in DNA methylation or some histone modifications in our examination of the *Commd1* promoter [27].

Our results implicate the existence of unidentified growing oocyte-specific factors for the establishment of methylation at maternal gDMRs. We have yet to determine whether these factors are proteins, RNAs, or chromatin structures. Future extensive proteomic, transcriptomic, and epigenetic analyses of the growing oocyte may elucidate the underlying mechanism for the primary establishment of methylation at the maternal gDMRs.

## Conclusions

We have found that *Commd1* was expressed and thus the *Zrsr1*-DMR was transcribed in growing oocyte during the establishment of methylation at the DMR. Our data demonstrate that the transcription through the *Zrsr1*-DMR is essential for the establishment of methylation at the DMR in the growing oocyte. Since methylation at the *Zrsr1*-DMR does not occur during embryonic development and postnatal growth, the mechanism of the DMR methylation is suggested to be oocyte-specific.

The imprinted paternal expression of *Zrsr1* is caused by the maternal methylation at the *Zrsr1*-DMR which represses the maternal allele expression. The paternal expression of *Zrsr1* results in predominant maternal expression of *Commd1*, likely caused by transcriptional interference between *Zrsr1* and *Commd1* on the paternal allele.

## Methods

### Generation of *Commd1*-*PA* mice

A truncation cassette was constructed by cloning three copies of the SV40 poly(A) signal from the expression vector pGFP-N1 (Clontech) into pT7Blue (Novagen). The truncation cassette was inserted at the genomic site 23 bp downstream of exon 1 using the gene-targeting method (Fig. 1a). The targeting construct was generated by homologous recombination with a truncation cassette clone and a mouse *Commd1* BAC clone (BAC RP24-216A32, BACPAC Resources) in *E. coli* as previously described [35]. The targeting construct contained the following mouse *Commd1* genomic sequences: a 5.2-kb 5′ sequence containing exon 1 and a 5.1-kb 3′-sequence containing a part of intron 1. An embryonic stem cell (ES) clone with the truncation cassette inserted in the precise genomic position was identified by Southern blotting and PCR analyses of genomic structure and was used to generate chimeric *Commd1*-*PA* mice. The *neo* gene in the targeting vector was flanked by FRTs and removed from the *Commd1*-truncated mice via Flippase expression. *Commd1*-*PA* mice in a C57BL/6 J (denoted B6) genetic background were obtained by backcrossing with wild type (WT) C57BL/6 J mice for five generations.

### Preparation of primordial germ cells, metaphase II oocytes, and blastocysts

Mouse female primordial germ cells (PGC) were prepared from Day 5, Day 10, and Day 15 female C57BL/6 neonates as previously described [36]. The metaphase II (MII) oocytes were obtained from superovulated six- to eight-week-old female mice using the procedure described by Nakagata et al. [37]. Blastocysts were prepared by in vitro fertilization (IVF) using MII oocytes from *Commd1*^PA/+^ B6 mice and sperm from 12- to 14-week-old CAG-Cre transgenic BALB/c mice. After cumulus–oocyte complexes had been coincubated with sperm for 3 h, fertilized oocytes were cultured to the blastocyst stage at 37 °C and 5% CO_2_ in humidified air for 96–120 h, as previously described [24]. CAG-Cre BALB/c mice were obtained from the RIKEN BioResource Center (RBRC No. RBRC06155). The CAG promoter drives ubiquitous expression of Cre recombinase in mice.

### DNA preparation and methylation analysis

Approximately 200 growing oocytes and MII oocytes were lysed in lysis buffer (0.5% SDS, 250 ng/µl proteinase K, 100 ng/μl yeast tRNA) at 37 °C for 60 min, and DNA in the lysate was bisulfite-converted by treating the lysate with the EpiTect Bisulfite Kit (QIAGEN, #59104). Recovered DNA was used for amplification of *Zrsr1*-DMR via nested PCR. Amplified DNA was cloned in pT7Blue T-vector (Novagen, #69820), and the resulting sequences were analyzed with BigDye Terminator v3.1 (Applied Biosystems, #4337455) on an ABI3130 sequencer (Applied Biosystems). Blastocyst DNA was prepared from the precipitate of ISOGEN II lysate during RNA preparation with ISOGENOME (NIPPON GENE, #318-08111). DNA from adult tissues was prepared using QIAamp DNA Mini Kit (QIAGEN, #51306). Blastocyst DNA was genotyped by genomic PCR, using primers on both sides of the truncation cassette (Fig EV2). DNA from blastocysts and tissues was also bisulfite-converted using the EZ DNA Methylation Kit (Zymo Research Corp., #D5001). PCR, cloning, and sequencing were performed as described for oocyte DNA.

### RNA preparation

RNA was prepared from approximately 100 growing and MII oocytes using the RNeasy Micro Kit (QIAGEN, #74004). For RNA preparation from blastocysts and adult tissues, samples were lysed with ISOGEN II (NIPPON GENE, #311-07361), and the cleared lysates were recovered by centrifugation according to the manufacturer’s instructions. Three blastocysts were pooled for RNA preparation, and the lysate was loaded onto a spin column from the RNeasy Mini Kit (QIAGEN, #74104). DNase I treatment, column wash, and RNA elution were performed according to the manufacturer’s instructions.

### Expression analysis

To analyze *Commd1* expression in oocyte and adult tissues, cDNA was synthesized with random primers (Takara, #3802) and reverse transcriptase (TOYOBO, TRT-101), and the *Commd1* cDNA was amplified by PCR with the primers Comm-F1 (exon 1) and Comm-R1 (exon 2). To analyze allelic expression, restriction fragment length polymorphism (RFLP) analysis was performed via NlaIII digestion of the amplified cDNA. Allelic expression of *Zrsr1* was analyzed by BigDye terminator sequencing of the cDNA amplified with the primers Zrsr-F1 and Zrsr-R1. No rs numbers were assigned to the single nucleotide polymorphisms (SNPs) that were used to discriminate between the C57BL/6 J and PWK alleles. Quantitative allelic expression analysis of *Commd1* in blastocyst and adult tissues was performed in triplicate by pyrosequencing with PyroMark Q24 (QIAGEN) in the AQ assay mode. The rs26846230 SNP was used to discriminate between the C57BL/6 J and BALBc alleles. Total expression levels were quantitated in triplicate using the StepOnePlus real-time PCR system with TaqMan Gene Expression Assay (Applied Biosystems) Mm00495837_s1 and Mm01239669_m1 for *Zrsr1* and *Commd1*, respectively. Actb (β-actin) mRNA was quantitated as an internal control using TaqMan Assay Mm00607939_s1.

### Primers

All primers used in this study are listed in Additional file 4: Table S1.

## Additional files

**Additional file 1: Figure S1.** RT-PCR analysis of *Zrsr1* expression in growing oocytes. RT-PCR was done with primers Zrsr-F1 and Zrsr-R1 using cDNAs in Fig. 1b. Growing oocytes were prepared from B6 female neonates at Day 5 (D5), Day 10 (D10), and Day 15 (D15) postpartum, and fully grown MII oocytes (MII) from B6 adult females. PC: positive control for RT-PCR using adult brain cDNA. MW: molecular weight marker. Two different cDNA batches were used for D5 RNA.

**Additional file 2: Figure S2.** Genotyping PCR of blastocysts carrying *Commd1*^*PA*^ and CAG-Cre transgene. **A** Schematic representation of PCR for three *Commd1* alleles, *Commd1*^*PA*^ (PA), *Commd1*^*DPA*^ (DPA) and *Commd1*^+^ (WT). **B** Electrophoresis of PCR products of nine blastocysts positive for *Commd1*^*PA*^ and CAG-Cre transgene among 24 blastocysts obtained from an IVF performed with oocytes from PA female mice and sperm from CAG-Cre male mice. Two blastocysts (#5, #9) contained small amount of undeleted truncation cassette. MW: molecular weight marker.

**Additional file 3: Figure S3.** Quantitative analysis of the allelic expression of *Commd1* in adult mice. The allelic expression was quantitatively analyzed by pyrosequencing. The levels of expression from the parental alleles are shown relative to the level of the paternal allele (1.0). Each sample was analyzed in triplicate. **A** Brains and livers from two WT adult F1 mice between B6 females and PWK males. **B** Brains and livers from three WT adult F1 mice between B6 females and BALBc males.

**Additional file 4: Table S1.** List of the primers used in this study.

## Abbreviations

cDNA: complementary DNA
DMR: differentially methylated region
mRNA: messenger RNA
PCR: polymerase chain reaction
RT-PCR: reverse transcription PCR
