# Supplementary figures and images for: Growing oocyte-specific transcription-dependent de novo DNA methylation at the imprinted Zrsr1-DMR
Source: Epigenetics Chromatin. 2018 Jun 6;11:28. doi: 10.1186/s13072-018-0200-6 (PMC5989421; doi:10.1186/s13072-018-0200-6)

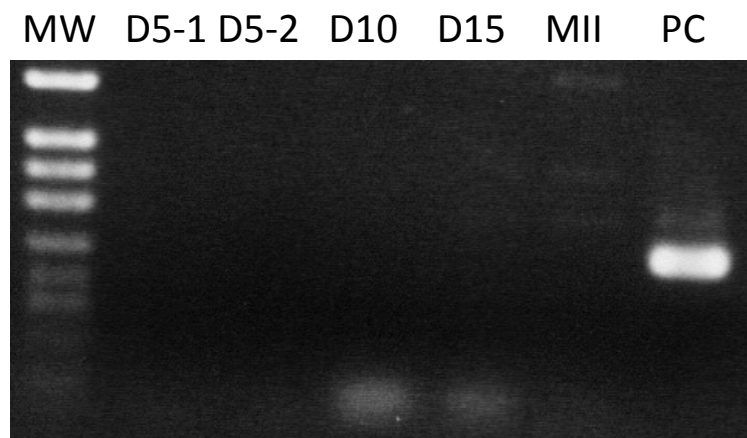

**Joh *et al.*, Additional file 1: Figure S1.**

Supplement: Supplementary file 1 — Additional file 1: Figure S1. RT-PCR analysis of Zrsr1 expression in growing oocytes. RT-PCR was done with primers Zrsr-F1 and Zrsr-R1 using cDNAs in Fig. 1b. Growing oocytes were prepared from B6 female neonates at Day 5 (D5), Day 10 (D10), and Day 15 (D15) postpartum, and fully grown MII oocytes (MII) from B6 adult females. PC: positive control for RT-PCR using adult brain cDNA. MW: molecular weight marker. Two different cDNA batches were used for D5 RNA. [file 13072_2018_200_MOESM1_ESM.pdf]

A

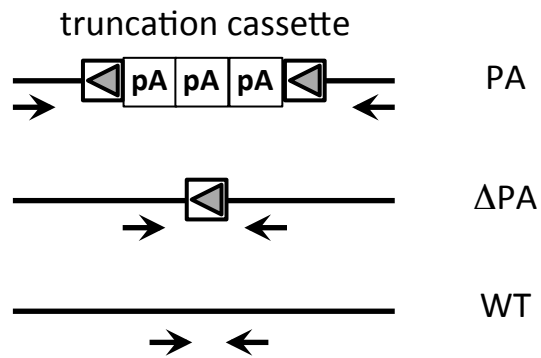

B

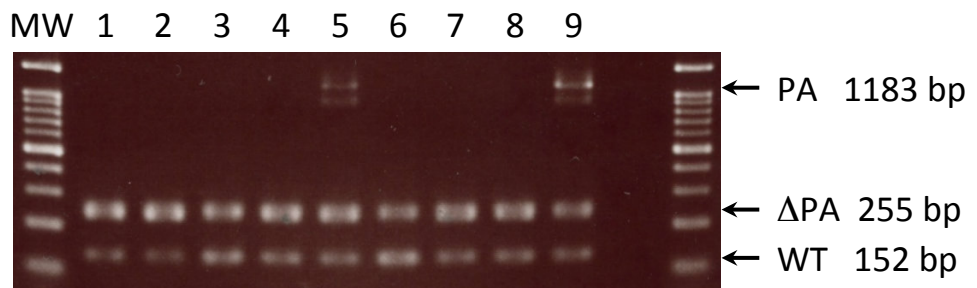

Joh *et al.*, Additional file 2: Figure S2.

Supplement: Supplementary file 2 — Additional file 2: Figure S2. Genotyping PCR of blastocysts carrying Commd1PA and CAG-Cre transgene. A Schematic representation of PCR for three Commd1 alleles, Commd1PA (PA), Commd1DPA (DPA) and Commd1+ (WT). B Electrophoresis of PCR products of nine blastocysts positive for Commd1PA and CAG-Cre transgene among 24 blastocysts obtained from an IVF performed with oocytes from PA female mice and sperm from CAG-Cre male mice. Two blastocysts (#5, #9) contained small amount of undeleted truncation cassette. MW: molecular weight marker. [file 13072_2018_200_MOESM2_ESM.pdf]

**A**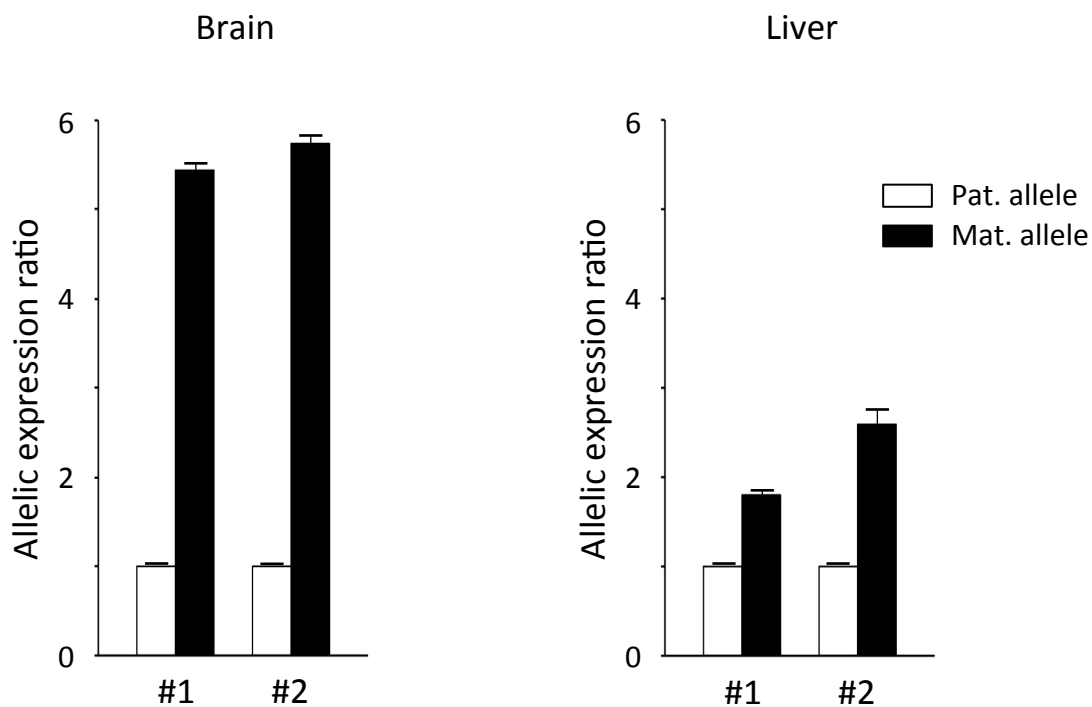**B**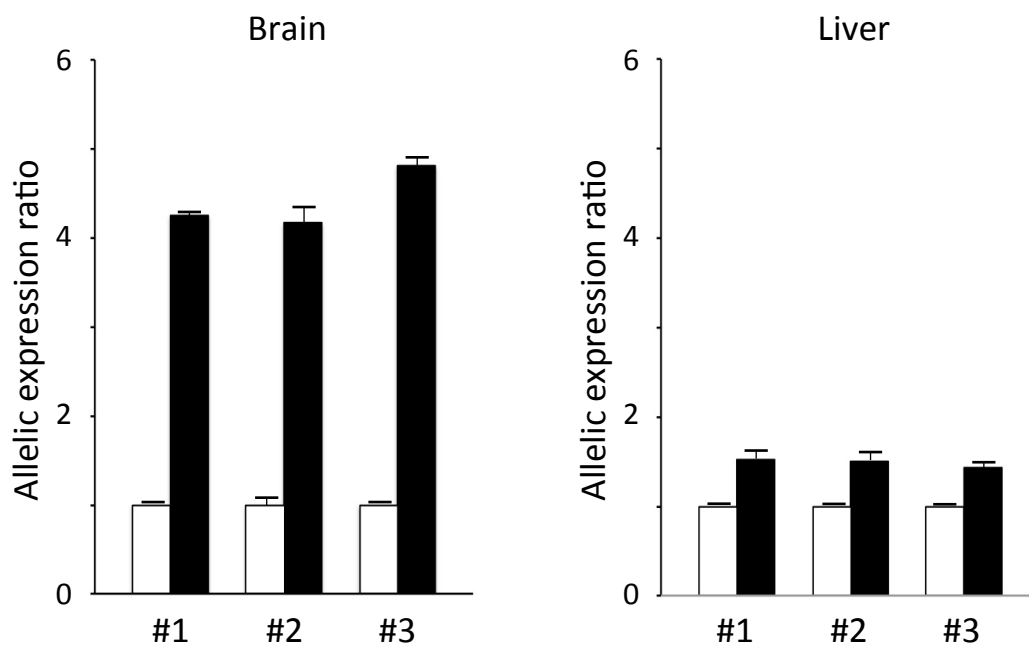

Supplement: Supplementary file 3 — Additional file 3: Figure S3. Quantitative analysis of the allelic expression of Commd1 in adult mice. The allelic expression was quantitatively analyzed by pyrosequencing. The levels of expression from the parental alleles are shown relative to the level of the paternal allele (1.0). Each sample was analyzed in triplicate. A Brains and livers from two WT adult F1 mice between B6 females and PWK males. B Brains and livers from three WT adult F1 mice between B6 females and BALBc males. [file 13072_2018_200_MOESM3_ESM.pdf]
